# Living with brain metastasis – a qualitative study of patients’ and family members’ coping strategies

**DOI:** 10.1080/17482631.2025.2555228

**Published:** 2025-09-01

**Authors:** Daniela Lillekroken, Asta Bye, Liv Halvorsrud, Tonje Lundeby

**Affiliations:** a Department of Nursing and Health Promotion, Oslo Metropolitan University, Oslo, Norway; b European Palliative Care Research Centre, Department of Oncology, Oslo University Hospital, Oslo, Norway; c Institute of Clinical Medicine, University of Oslo, Oslo, Norway

**Keywords:** Brain metastases, coping strategies, family caregivers, individual interviews, thematic analysis

## Abstract

**Background:**

Brain metastases are a serious complication of advanced cancer, often impairing patients’ neurological function, quality of life, well-being, and prognosis. They also place a heavy emotional burden on family members. Coping strategies play a crucial role in reducing stress, supporting emotional well-being, and adapting to these challenges. Gaining insight into how patients and families cope is important for developing person- and family-centred interventions.

**Aim:**

To explore similarities and differences in coping strategies between patients with newly diagnosed brain metastases and their family members from diagnosis to four months thereafter.

**Methods:**

The study has a qualitative, exploratory, longitudinal design. Between 2019 and 2021, 81 individual interviews were conducted with patients and their family members. Data were analysed using a secondary thematic analysis.

**Results:**

The analysis revealed two main themes: Willpower – taking control of the mind, and Reframing life – here-and-now versus long-term plans. While patients exhibited willpower and took control over their minds, focusing on the present moment, family members emphasised reframing life by balancing the immediate needs and information seeking with long-term planning.

**Conclusions:**

Patients and family members employ distinct coping strategies. Recognising these differences provides a foundation for tailored, person- and family-centred interventions aimed at improving quality of life.

## Introduction

Brain metastases (BMs) are a serious complication of cancer linked with a high symptom burden, substantial neurocognitive impairment, and a poor prognosis (Fares et al., 2025). About 10%–30% of all cancer patients develop BMs, and many experience symptoms such as headaches, fatigue, nausea, and cognitive decline (Mitchell et al., 2022). These physical and cognitive effects of BMs may cause emotional stress, as well as social and economic challenges, not only for the patients but also for their families, who face an uncertain future (Maqbool et al., 2017; Unsar et al., 2021). Stress significantly threatens an individual’s psychological well-being (Pinquart & Duberstein, 2010), especially when coping with BMs. Psychological well-being refers to a person’s feeling of happiness, satisfaction with life, and capacity for personal growth (Dhanabhakyam & Sarath, 2023). It is a vital aspect of mental health and is generally linked with higher happiness and life satisfaction, while inversely related to stress and depression (Pinquart & Duberstein, 2010).

End-of-life care for patients with BMs has been extensively researched (Greer et al., 2022; Harrison et al., 2024; Kaiser et al., 2020; Koekkoek et al., 2023; Yip et al., 2021). Recently, numerous intervention studies have been carried out to improve coping strategies among patients with cancer and their family members (Lambert et al., 2025; Ma et al., 2025; Yildirim et al., 2024), thereby demonstrating that enhancing psychological support in oncology care has become a growing research and clinical priority, with evidence indicating that such interventions may effectively strengthen coping mechanisms in both patients and their families. However, limited attention has been paid to how patients and their families cope with a BMs diagnosis and living with the condition. In this context, coping involves thoughts and behaviours used to manage stressful situations (Folkman & Moskowitz, 2004). When receiving a serious diagnosis, people adopt different coping strategies to handle the new situation, depending on factors such as age, gender, socioeconomic status, and the nature of the stressful scenario. According to Folkman and Moskowitz (2004), the four most common coping strategies are: (i) problem-focused coping, which addresses the cause of distress by actively tackling the issue and includes seeking information, planning, exercising restraint, and suppressing distractions; (ii) emotion-focused coping, which aims to reduce negative emotions related to the problem through positive reframing, acceptance, reliance on religious faith, and humour; (iii) meaning-focused coping, which involves using cognitive strategies to interpret and manage the significance of a situation; and (iv) social coping (support seeking), which helps individuals alleviate stress by reaching out for emotional or practical support from their social networks.

Several studies demonstrate that younger people often cope through hope, while older individuals tend to choose resignation (Hernández et al., 2019; Kang & Son, 2019; Yi & Kase, 2023). Women often use positive reframing or religion, while men usually rely on humour (Oppegaard et al., 2020).

Existing research on coping strategies in patients with cancer emphasizes the crucial role that psychological factors play in how individuals adapt to illness. For example, Pruyn (1983) discussed the significant emotional challenges faced by cancer patients, outlining stressors such as uncertainty, negative feelings, loss of control, and threats to self-esteem. The study also explored various coping strategies, including seeking information, support, and comfort, as well as attribution, impulsive actions, confrontation avoidance, and active coping behaviours.

Miller et al. (1996) found that optimism and coping strategies influence psychological adjustment, even when previous adjustment and physical functioning are considered. Optimism is associated with greater well-being and less distress, while escape-avoidant coping increases distress, and accepting responsibility decreases well-being. Rachel et al. (2022) also emphasized the impact of coping on caregiver satisfaction, noting that venting significantly affects one dimension of the Caregiving Satisfaction Scale.

Recent studies have shown that individuals diagnosed with conditions such as cancer, whether with or without metastases, along with their family members, often adopt different coping strategies to manage the illness. Ismael et al. (2024) investigated the coping strategies of caregivers of children who survived paediatric cancer in Jordan, revealing that caregivers frequently rely on passive coping mechanisms, including religion and acceptance, to handle daily stressors. Harel et al. (2025) examined the relationship between coping styles (approach and avoidance), emotional processing (emotional awareness and acceptance), and cancer-related symptoms, demonstrating that accepting emotions is negatively associated with depression, while avoidance coping is positively associated with depression, cancer-related fatigue, and pain interference. Mardani et al. (2023) explored fear of cancer recurrence and coping strategies among prostate cancer survivors, identifying approaches such as problem-solving and emotional regulation. In a subsequent study, they explored coping with sexual dysfunction in Iranian prostate cancer survivors, highlighting the influence of cultural norms and personal resilience (Mardani et al., 2024). More recently, Mardani et al. (2025) conducted a systematic review and thematic analysis of 16 qualitative studies on coping with fear of recurrence in breast cancer survivors. They found that patients use both adaptive strategies, such as seeking support, improving health behaviours, and building resilience, as well as maladaptive strategies, such as avoidance and emotional detachment. Although these studies collectively demonstrate the diversity and complexity of coping strategies adopted across different patient groups, diagnoses, and cultural contexts, emerging evidence underscores that coping is not a static process but evolves as patients and family members navigate various phases of the cancer trajectory. For instance, Wei et al. (2023) observed shifts in coping strategies and spiritual well-being across the chemotherapy timeline among colorectal cancer patients, highlighting the dynamic nature of coping in response to treatment-related stressors. Similarly, Luu et al. (2024) reported dynamic patterns in coping among advanced cancer patients, noting that while some strategies remained stable, others changed in relation to clinical developments and personal circumstances.

Despite advances in oncology and palliative care, research specifically focusing on the lived experiences of patients with BMs remains limited. Much of the existing literature tends to emphasize clinical outcomes, symptom management, or the neurocognitive effects of BMs. The lack of comparative insight into how patients and their family members cope with BMs over time creates a significant gap in healthcare professionals’ understanding of how coping develops within the patient—family unit. To the best of our knowledge, no previous qualitative studies have explored the similarities and differences in coping strategies between patients with newly diagnosed BMs and their family members. Therefore, further research is needed in this area.

## Methods

### The study’s aim and design

This study aims to explore potential similarities and differences in the coping strategies of patients diagnosed with BMs and their family caregivers from the time of diagnosis through the subsequent two and four months. The study employs a qualitative, exploratory, longitudinal research (QLR) design, which involves repeated data collection that focuses on the temporality (i.e., time and change) of a phenomenon (Audulv et al., 2022). We employed a QLR design to capture the progression of BMs and the patients’ and their family members’ coping strategies.

### Study setting and recruitment

Participant inclusion started in 2019 and ended in 2021. Purposeful sampling was used to identify patients diagnosed with BMs. The patients and their family members were recruited from a prospective observational study (NCT03346655) conducted in Norway. All patients with a first diagnosis of BMs at three participating hospitals in the South-Eastern Norway Regional Health Authority were considered eligible. Those who consented to participate in the “BrainMet” research program at Oslo University Hospital and met the study’s inclusion criteria received verbal information about the study’s aim from the principal investigator or a research nurse.

Inclusion criteria—patients:

- A verified diagnosis of a solid tumour
- BMs verified by computed tomography, contrast-enhanced magnetic

resonance imaging, or surgical biopsies

- Aged ≥18 years
- Be able to comply with the overall study procedures, physically and cognitively
- Be able to provide written informed consent after information is provided in Norwegian
- Willing to participate in two or three in-person interviews

Exclusion criteria—patients:

- Primary brain tumour
- Previous diagnosis and/or treatment of BMs
- Cognitive, mental, or physical impairment preventing study compliance
- Unable to produce written informed consent after receiving information in Norwegian
- Death or deteriorating health condition prevented further participation in the study

At the time of inclusion, patients were asked whether the interviewers could invite the persons they defined as their primary family members or next of kin to participate in the study. If the patients consented and, from a practical point of view, if possible, family members were informed about the study and asked to participate by the principal investigator of the main study or a research nurse.

Inclusion criteria—family members:

- Cognitively able and willing to take part in the study
- Possesses sufficient language skills to understand the verbal and written information
- Able to provide written informed consent
- Aged ≥18 years

Exclusion criteria—family members:

- Unable to provide written informed consent after receiving information in Norwegian

Alongside the interviews, participants provided self-reported demographic information before the first interview to help characterize the sample, evaluate its diversity, and assess the potential transferability of the findings to other contexts.

### Data collection

The data were collected by conducting individual interviews (Knott et al., 2022). Individual interviews were chosen to gain deeper insight into the personal experiences, coping strategies, and emotions of patients with BMs and their families. Due to the sensitive and complex nature of life-limiting illness, interviews offer a private, supportive setting for open sharing, which group formats may not allow because of emotional or cognitive challenges linked to BMs. This method also enables researchers to explore how each person uniquely perceives and manages the illness, which is especially important when capturing both patients’ and family members’ perspectives. In addition, individual interviews facilitate a more flexible and responsive approach to data collection, allowing the interviewer to adapt to the cognitive or emotional needs of the participants, which is essential in the context of serious illness. Individual interviews allow individuals to explain, in their own words, how they understand and interpret the world around them (Knott et al., 2022).

Between 2019 and 2021, 81 interviews were conducted at the hospital in connection with scheduled appointments. The participants were interviewed three times: at the time patients received a BMs diagnosis (T0), two months later (T1), and four months later (T2). Family members were interviewed simultaneously with the patients, but in separate rooms. This longitudinal approach was chosen to capture the dynamic and evolving nature of their emotional, cognitive, and practical experiences over time.

Due to the SARS-CoV-2 virus pandemic and social distancing guidelines, some interviews were conducted by phone. These interviews were conducted by two healthcare professionals employed at the hospital where the patients received the BMs diagnosis. These interviewers are not involved as authors in the present study. Both interviewers conducted the first two interviews to ensure similar performance between the interviewers. After this, each patient and their family member were interviewed by one interviewer.

The interviews were conducted in Norwegian and guided by an interview guide developed based on research literature. The interviews lasted approximately 35 minutes and were digitally recorded. The main themes covered in the interviews, along with the questions asked, are presented in Tables 1 and 2. Each new topic began with an open-ended question aligned with the research focus, followed by follow-up questions.

**Table 1.** Interview guide – themes and questions asked during interviews with patients.

The time of the interview	Interview with patients
When the BMs diagnosis was received (T0) The themes discussed at T0: – When the diagnosis was received – The content of the consultation, including shared decision-making – Daily life – Concerns – Other matters	Can you please describe how it was to receive BMs diagnosis? What did you and the doctor speak about when you received the diagnosis? How have you managed/functioned in daily life recently? Have you experienced any symptoms? Which ones? What is most important to you right now? Is there anything else, in addition to what we have discussed today, that you would like to bring up?
Two months later (T1) The themes discussed at T1: – Daily life – Concerns – Shared decision-making – Anticipated care needs – Other matters	How have you managed/functioned in daily life over the past week? Have you experienced any symptoms? If so, which ones? What is most important to you right now? Have you received any treatment for brain metastases? If so, how were the different treatment options presented to you? This is a serious diagnosis. Has anyone spoken with you about the type of treatment, care, and support you might need at a later stage? Is there anything else you would like to bring up, in addition to what we have discussed today? How has it been for you to participate in this interview?
Four months later (T2) The themes discussed at T2: – Daily life – Concerns – Shared decision-making – Anticipated care needs – Other matters	How have you managed/functioned in daily life over the past week? Have you experienced any symptoms? If so, which ones? What is most important to you right now? Have you received any treatment for the BMs? If so, how were the different treatment options presented to you? This is a serious diagnosis. Has anyone spoken with you about the type of treatment, care, and support you might need at a later stage? Is there anything else you would like to bring up, in addition to what we’ve talked about today? How has it been for you to participate in this interview? You and your family member have been interviewed separately over the past weeks, but have been asked questions related to the same themes. Can you describe what this experience has been like for you?

**Table 2.** Interview guide – themes and questions asked during interviews with family members.

The time of the interview	Interview with family members
When the BMs diagnosis was received (T0) The themes discussed at T0: – When the diagnosis was made – The content of the consultation, including shared patient/family member decision-making – Concerns – Other matters	Can you please describe how you found out that your family member was diagnosed with BMs? Did you discuss different types of treatment with the doctor? What is most important to you right now? Is there anything else, in addition to what we have talked about today, that you would like to bring up? How has it been for you to participate in this interview?
Two months later (T1) The themes discussed at T1: – Daily life – Concerns – Family members’ experiences with healthcare services after receiving the BMs diagnosis – Other matters	How do you feel your life has been affected since your family member was diagnosed with BMs? What is most important to you right now? All in all, how would you describe the experiences you have had with the healthcare services since your family member received the diagnosis? Is there anything else you would like to bring up, in addition to what we’ve talked about today? How has it been for you to participate in this interview?
Four months later (T2) The themes discussed at T2: – Daily life – Concerns – Family members’ experiences with healthcare services after receiving the BMs diagnosis – Other matters	How do you feel your life has been affected since your family member was diagnosed with BMs? What is most important to you right now? All in all, how would you describe the experiences you have had with the healthcare services since your family member received the diagnosis? Is there anything else you would like to bring up, in addition to what we’ve talked about today? How has it been for you to participate in this interview? You and your family member have been interviewed separately over the past weeks, but have been asked questions related to the same themes. Can you describe what this experience has been like for you?

### Data analysis

All interviews were transcribed verbatim by professional transcriptionists and checked for accuracy. For this study, the analysis was conducted by four researchers with extensive experience from qualitative research, the authors of this paper, who were not involved in the original data collection. To analyse the data, thematic analysis, as recommended by Braun and Clarke (2006), was employed. The data were manually analysed, and an open coding approach was used.

This study presents findings from a secondary analysis of qualitative interviews conducted with patients newly diagnosed with BMs and their family members as part of a comprehensive research program, “BrainMet,” conducted at Oslo University Hospital. Secondary analysis involves re-examining existing data to address new research questions or gain more profound insights beyond the original study’s purpose (Kelly et al., 2024). This approach allows researchers to maximize the utility of qualitative data, especially when exploring perspectives across different participant groups.

While the primary analysis aimed to explore only the patients’ individual experiences of receiving and living with BMs, findings presented elsewhere, this study focuses on analysing all interview data from both patients and their family members to identify similarities and differences in coping strategies between these two groups.

Data were analysed holistically across time points to capture overarching themes over time across participant groups. For each participant, all available interviews were read and analysed together, enabling researchers to trace changes and continuities over time. This was first done for patients and subsequently for family members. This approach allowed researchers to capture developments over time while maintaining a focus on participants’ overall experiences with the coping strategies they employed.

The analysis was conducted in six steps, following the thematic analysis approach outlined by Braun and Clarke (2006). First, the researchers familiarized themselves with the data. Each researcher was responsible for reading, rereading, and analysing a selected number of transcripts (five interviews with patients and five interviews with family members). Second, the initial codes were generated by open coding. As advised by Braun and Clarke (2006), not every statement was coded; only those related to the study’s aim. A search for subthemes in the codes was conducted across the whole dataset to prevent the same codes from being included in multiple subthemes. In the third step, all four researchers met and discussed the suggested subthemes. These discussions led to a review and eventual alteration of the subthemes. In the fourth step, the subthemes that described similar content were defined and labelled. All controversies were discussed between the researchers until a decision was made. Finally, in the fifth step, these subthemes were grouped once again to develop the final axes based on conceptual relevance, resulting in two main themes and eight subthemes. The resulting list of subthemes and themes was reviewed and discussed by all authors to enhance the trustworthiness and credibility of the data analysis and the study findings.

A total of 81 individual interviews generated a large volume of data. To reach saturation, the researchers cross-checked codes and themes across data, confirming that no new information emerged; thus, theoretical saturation was achieved (Saunders et al., 2018). An example of the analytical process that generated one of the themes is provided in Table 3.

**Table 3.** Example of coding tree.

Example of quotations/codes	Subtheme	Theme
Don’t think the “worst case” (FM1, I3) Living a good life while I still have it (FM3, I3) A need for a normal life (P5, I1) Optimism (P2, I1&3) Feeling safe and trusting the doctors and the treatment (P4, I1) Wishing to go back to work (P5, I2) Being socially active (P4, I1)	Choosing to think positively Maintaining hope—there is a future Relying on others—believing and trusting in healthcare professionals and the treatment process Keep going—avoidance and floating on the surface	Willpower—taking control of the mind

### Ethical approval

The study was conducted in accordance with the principles of informed consent, potential consequences, and confidentiality outlined in the World Medical Association’s Declaration of Helsinki (World Medical Association, 2025). Ethical approval was granted by the Regional Committee for Medical and Health Research in Ethics (REK 2018/800 REK South-East B). Authorisation for data storage was provided by the data protection officer at Oslo University Hospital, in accordance with institutional procedures. All participant data were de-identified and stored on a password-protected computer. Only authorized researchers had access to the data. Current institutional guidelines for the storage, transmission, and disclosure of patient information were followed, as outlined in existing rules and regulations for research data storage at Oslo Metropolitan University and Oslo University Hospital.

Cognitive impairment is common in patients with BMs and affects their capacity to consent (Triebel et al., 2015); therefore, no patient was included if there were questions about their capacity to consent. Before the interviews, all participants were informed about the study and asked to sign a consent form that outlined the confidentiality of the interviews, the fact that their answers would not influence the services they received, and their right to withdraw from the study at any time. This information was also explained verbally before the interviews commenced. Informed consent was obtained from all participants prior to the interviews, and written consent was obtained. Participants were reminded of the value of their input and given the opportunity to ask questions or express any concerns before participating. None of the participants chose to do so. The participants did not receive any economic or other material benefits for taking part in the study; however, their involvement was greatly appreciated, as it contributed to advancing knowledge in the field of oncology and supporting future improvements in healthcare practice and policy.

Following each interview, the researcher asked the participants about their experience participating in the study to ensure the interview had not caused any distress. Moreover, during the interviews, participants were given the opportunity to pause, take breaks, or end the interview at any time. Interviewers provided contact information for support services if needed.

### Rigour of the study

The four criteria of credibility, dependability, confirmability, and transferability, described by Lincoln and Guba (1985), were used to establish the study’s rigour and trustworthiness. The interview transcripts were independently analysed and interpreted, and the findings were discussed, compared, and corroborated in the research group. Discussion of the findings aimed to reach consensus and establish trustworthiness, thereby enhancing dependability. Inter-rater reliability was evident in the comparison of findings between all four researchers, as well as in the explicit notation of coding decisions and the creation of clear definitions in the codebook (Morse, 2015). The sampling strategy was purposeful, providing a maximum variation of different genders, ages, and experiences of both patients and family members to enhance credibility. Detailed information on the recruitment and inclusion criteria of the participants was also provided to enhance the transferability of the findings to similar contexts. The quotations in the findings section illustrated the patients’ and their family members’ statements, thus enhancing confirmability. For the purposes of the manuscript, illustrative quotes were translated into English. The translation was carried out by the first author, and to ensure accuracy, it was cross-checked by the co-authors and a proofreading editing service. The quotations are presented fairly and were recognized as clinically relevant to the setting (Lincoln & Guba, 1985).

## Results

### Characteristics of the participants

A heterogeneous, purposeful sampling procedure was employed, focusing on age, gender, and primary cancer diagnoses, to obtain a sample that reflects variations in cancer diagnoses and other characteristics. A total of 38 participants, consisting of patients (*N* = 20) and their family members (*N* = 18), were included. Unfortunately, because one of the audiotapes was accidentally deleted, only 17 interview transcripts with family members were included in the analysis.

The ages of the patients ranged from 43 to 81 years, and 12 were women. Eleven patients had a university education, six had a high school education, and one had an elementary education. The education level of the two patients was unknown. Primary cancer diagnoses included breast cancer (*N* = 5), lung cancer (*N* = 6), gastrointestinal cancer (*N* = 2), rectal cancer (*N* = 2), and malignant melanoma (*N* = 5). At the time of the interviews, all patients were undergoing treatment for BMs, such as surgery (*N* = 5), stereotactic radiotherapy (*N* = 5), whole-brain radiotherapy (*N* = 8), and systemic therapy (*N* = 1). Treatment information was unavailable for one patient. Due to health deterioration or death, only nine patients completed all three planned interviews. Three patients participated in two interviews, and eight in a single interview. At the time of BMs’ diagnosis, most patients had experienced disease progression long after their primary cancer. Detailed staging information was not consistently available.

The family members ranged in age from 34 to 86 years, with six being women. The majority (*N* = 13) were spouses or partners, two were siblings, and two were sons.

Overall, the analysis revealed distinct coping strategies between patients and their family members. Two main themes of coping strategies were identified: (i) Willpower—taking control of the mind, and (ii) Reframing life—here-and-now versus long-term plans. Four subthemes were identified under each theme. The patients coped more often by focusing on positive thinking, maintaining their everyday lives, trusting the treatment process, and taking one day at a time. Family members coped by seeking information, planning the future, and organizing family life.

In the following section, the findings are presented, accompanied by quotations from the participants’ statements that illustrate each theme. Each statement concludes with a number representing the code for each patient (e.g., P1), family member (e.g., FM1), and interview number (e.g., I1).

### Willpower—taking control of the mind

By adopting different cognitive coping strategies, the patients, as well as their family members, managed to reduce distress and minimize the psychological impact of being diagnosed with BMs. This theme is supported by four subthemes: (i) Choosing to think positively, (ii) Maintaining hope—there is a future, (iii) Relying on others—believing and trusting in healthcare professionals and the treatment process, and (iv) Keep going—avoidance and floating on the surface.

### Choosing to think positively

Although both patients and their family members were concerned about the BMs and whether the treatment would be effective, they addressed these concerns in different ways. Most patients expressed that they did not want their worries to take over their lives and preferred to maintain a positive attitude. One of the patients said:

I choose to be optimistic, and then I choose to think that science has come a long way. I believe in thinking positively instead of going downhill. I believe it can also have an impact on overall health. It’s my … that’s what I have chosen to believe … (P21, I1)

Another patient adopted a positive attitude as a way of living, which, for him, was imperative when it came to coping with the diagnosis. He said:

I can tell you one thing … that door to anxiety is closed, and it’s locked. And that door to “this you can die of, … this can be life threatening for you … ,” that door … I’ve closed, […], and then I think only of the “best-case scenario” all the way … and that’s how I’m made, no matter what … (P3, I1)

Although thinking positively was not a prevalent coping strategy among family members, some highlighted the significance of upholding a positive attitude. One family member admitted that, although it was tough to receive bad news about his wife’s BMs, he was aware that “we shouldn’t think of the worst-case scenario unless there’s a reason” … (FM1, I3)

### Maintaining hope—there is a future

Several patients, as well as some of their family members, used hope as a positive future-oriented coping strategy. The patients allowed themselves short periods in which they acknowledged doubts about the future, but most managed to reinvigorate their hope. Hope was used to strengthen the feeling that there is a way out of difficulties. For many patients, hoping for disease regression or cure was an essential coping strategy, because not all patients and family members may have fully understood the severity of the BMs condition intellectually or accepted it emotionally, and many were convinced that the treatment would succeed. One of the family members acknowledged that for him, it was important to cling to hope:

That there’s a plan to be able to keep hope and know that this is … that there’s something you can do … (FM1, I1)

Although survival was the primary concern for many, some patients redefined their future life beyond merely counting years. As a coping strategy, they chose to see possibilities instead of limitations and to be aware of how they perceive reality in terms of hope for the future. However, while acknowledging that there is a future, some patients raised the question of what that future represents:

But then I lose faith sometimes, … and then it comes back, and I hope that things will go well … it takes a long time … it can take up to a year before things are back in place. So, I’m looking forward to Wednesday when I go for a check-up to hear a bit more about how things are going and if the prognosis is moving forward … [in a positive way] (P19, I2)

For another patient, hope was tied to life after treatment, healing, and recovery:

… that I, at least, have several relatively normal years ahead and that I have plenty of time with my family in the future. That’s what I’m hoping for, and that it won’t be too tiring with too many chemotherapy treatments and things like that … (P9, I1)

### Relying on others—believing and trusting in healthcare professionals and the treatment process

All patients reported receiving medical treatment and expressed belief in its effectiveness, expecting favourable outcomes. However, most patients and their family members were also aware of their limited control over the illness trajectory and treatment outcomes. As a coping strategy, several patients chose to place their trust in healthcare professionals, believing that “they’ll be able to fix me” (P3, I1). They viewed healthcare professionals as essential in maintaining their optimism for recovery. One patient reported that she felt well taken care of by a highly competent professional team:

I feel safe with the medical team … because I feel that it’s as good as it can be … I think there’s incredible warmth and care … and … simply, it’s good to be here … (P4, I1)

Another patient expressed gratitude for being admitted to the hospital and for healthcare professionals being open and informing her about the treatment, as well as for including her in the decision-making process:

Yes, I received excellent information to help me make that decision with confidence. That’s something I want to say. And, of course, I hope that many others also get to experience … [name of the hospital]—it’s great! Skilled people, the doctors there … Professional and excellent! (P20, I1)

Family members also expressed positive experiences with the healthcare services:

… I feel that they care about me, too … I have to say that my experience with the healthcare services has been very positive. I get the impression that the people working in there … [at the hospital] care about the person, not just the profession … (FM2, I2)

However, when the situation was almost unbearable, some family members felt that they needed more support from the healthcare services, as one of them said, “I’ve considered several times whether I should start group therapy …” (FM3, I1)

### Keep going—avoidance and floating on the surface

For most patients, it was essential to stay focused, meaning to concentrate on the present moment. Many patients reported consciously choosing to disregard problems that might arise in the near future. Some actively avoided thinking negative thoughts, as one of them conveyed in the following quotation:

I’m a rational person, so I try to push as much as possible in front of me … so, I instead choose to take it as it comes … (P1, I1)

Feeling physically well, along with optimism and positive thinking, helped the patients keep going. According to one of them, this was a strategy to avoid being overwhelmed by negative thoughts and emotions, helping them to distance themselves emotionally from reality and lessen their concerns about the future. One of the patients reported:

In my world, I’m healthy … I don’t know if I push it under the rug or if I push it in front of me, or what I’m doing, but at least I define myself as healthy … (P3, I2)

The avoidance strategy was common among the patients and less frequent among their family members. One family member described focusing on the good times while at the same time being aware of an uncertain future:

Yes, ever since she received the second treatment, it seems that it’s going in a certain direction … And now she’ll have another treatment in June, and then … we’ll see. Because I understand that she might not recover from this, or most likely won’t, therefore, it’s essential to have such good periods as we’re having now … (FM21, I3)

### Reframing life—here-and-now versus long-term plans

This theme highlights the importance for most patients and their family members to redefine the course of their lives, both as individuals and as a family unit, and is supported by four subthemes: (i) Seeking relevant information, (ii) Maintaining everyday life, (iii) Supporting and protecting one another, and (iv) Adapting to a life with uncertainty.

How the study participants reframed their lives differed between patients and family members. Many patients concluded that it was necessary to adopt a kind of *everyday pragmatism* and spoke about the practical ways in which they engaged with aspects of living with BMs and how it influences their lives. Most patients admitted that they had to reframe their lives, shifting from thinking, planning, and executing long-term life plans with and for the family to planning each day, hour by hour, based on their daily circumstances. Many of the family members expressed a need to make long-term life plans; they needed information about how to plan their future and organize their daily lives.

### Seeking relevant information

While some patients coped by using avoidance, some family members took the opposite approach. For many, researching survival rates, timelines, and methods to ensure the patient had good periods became a crucial strategy, not only to stay informed but also to maintain the family’s stability amid uncertainty. Especially for family members, seeking relevant information functioned as a defence mechanism by shifting the focus away from uncertainty. Staying engaged, rather than passively waiting, helped them concentrate on treatment goals and activities that enhanced their well-being. Both patients and family members sought to understand the BMs diagnosis and treatment options as a way of coping. Family members, however, took a more active role in seeking information and expressed a greater need for it. They used the internet and other sources significantly to gain information, as one family member said:

… because I’ve taken on the role of doing research and familiarising myself with things, as she hasn’t had the energy for it at times … (FM1, I2)

While family members took a more proactive approach, actively seeking additional details, the patients were generally content with the information provided by healthcare professionals. Furthermore, they relied on friends or other family members who had medical backgrounds. One of the patients shared her experience:

I have a sister who’s a doctor, so we talk a lot together. And she said that research has come a long way … And then I chose to rely on her knowledge, and I believe she knows a great deal about it … (P21, I1)

However, both patients and their family members had different information needs at various stages of the illness. While patients generally wanted to gain information about the present, family members wished for more detailed information about the future and what they should plan for.

### Maintaining everyday life

Another way to cope with the severity of the diagnosis and to distract themselves from negative thoughts was, for most of the patients and their family members, to keep themselves busy by working and/or doing whatever they could do around the house. According to one family member, his wife had a good period and “ … she helped shovel gravel” (FM21, I3).

For those who still had children under the age of 18, an essential coping strategy was to provide a sense of continuity in the family by dropping off and picking up their children at school, being together as much as possible as a family, and travelling on holidays. These participants described themselves as being “more than the illness,” and for them, it was important to maintain family functioning and have a sense of ordinary everyday family life. Some other patients of senior age were also concerned about keeping their physical activity, meeting family members or friends, and engaging in the activities and hobbies they enjoyed before their diagnosis. One of the patients said:

I feel privileged to have been able to function socially during this time. It’s almost unbelievable … I’ve been with friends and family, and I did a little bit of everything … (P2, I1)

To maintain everyday life despite the challenges of diagnosis and treatment, most family members, like the patients themselves, tried to prioritize their social lives, as one family member said:

I have a very good network, and I have spoken with them a lot, with my siblings, a sister, and one good friend … (FM3, I2)

### Supporting and protecting one another

During the interviews, some patients described intentionally shielding their family members from the burden of their health problems and treatment side effects, therefore minimizing the emotional impact on their loved ones, maintaining independence, and finding comfort in the presence of others as one way to cope.

One of the patients acknowledged that he did not tell all details about his illness, treatment, and outcomes or what he discussed with healthcare professionals to his wife to protect her because, as he said:

I think it’s going well, but I have a challenge with my wife, who actually takes this more seriously than I do …. (P3, I2)

Many patients sought to maintain a sense of normalcy and independence when going out or on hospital controls, as a way to cope. However, although most patients did not always ask for support, they recognized the importance of the support offered by family members, healthcare professionals, or friends and colleagues, and they were grateful when others acknowledged their needs. One of the patients shared his thoughts:

It was probably almost crucial for me that she [the wife] was with me [during treatments], because there was something about having someone to lie close to. Because that’s all you can handle … (P4, I1)

The findings from the interviews with family members revealed that they took on a protective role towards the patient, which helped them cope with the challenges associated with the patient’s diagnosis. Some family members took an action-oriented approach, actively participating in meetings with the patient and healthcare professionals to support the patient. As a way of coping, many family members chose to stay engaged by providing practical support, such as driving the patient to medical appointments, cleaning the house, shopping for them, preparing meals, and offering emotional support through conversation when needed. One participant said:

What I have taken mainly responsibility for is to help her with transport here and there and the purchase of food and things like that, when necessary … (FM2, I2)

Many family members mentioned that, from time to time, coping with the whole situation was tiring and that being a supportive partner was very challenging. Some also mentioned their own need for support to cope with the problem themselves. One family member said: “I would like to have somebody to talk with … ” (FM1, I3)

### Adapting to a life with uncertainty

Depending on the patients’ state and the treatment’s outcomes and/or side effects, coping fluctuated during the illness’s trajectory. When the patients felt well, family members believed they could more easily cope with the whole situation. Despite the uncertainty surrounding the illness trajectory, it was important for the patients and their family members to continue living their lives. Therefore, they had to adapt to life’s uncertainties. Both patients and family members navigated this process by selecting various strategies based on their perceptions of the current situation and different aspects of their lives. One of the family members, who used to make long-term plans and organize family life, revealed:

I’m living more in the present now, and less like … thinking long term! We’re at the here-and-now, in a way … I guess I’ve become much better at thinking in the present. I looked forward to things before, making long-term plans … Now, I don’t dare make so many plans (FM1, I1)

The same family member felt doubts and recognized that there were challenges with keeping everyday life similar to life before the diagnosis. He admitted that from time to time, his life was put on hold, and he felt that although “everyday life goes on its own … there’s something that puts life on hold” (FM1, I2). Moreover, he felt the stress because “ … I have to live with it daily” (FM1, I3), and that feeling was challenging to cope with.

Despite the serious diagnosis, both the patients and their family members realized that they had to go on with their lives. For several patients, this feeling was a way out of difficult situations. Their behaviour could foster feelings of coping well and controlling the situation, despite uncertainty. One patient said: “Yes, I have an overarching grip on my life, and I feel good about it!” (P2, I2).

For family members, they had to find pragmatic ways of coping more effectively when needed, such as adapting their lives to and prioritizing dealing with everyday challenges, as one of the family members stated:

There are many more practical tasks for me, and that only means that I have to prioritise differently …

(FM5, I3)

## Discussion

The study aimed to explore potential similarities and differences in coping strategies between patients diagnosed with BMs and their family caregivers from the time of diagnosis and the subsequent two and four months. Overall, the analysis revealed that, over time, the patients and their family members adopted distinct adaptive (positive) coping strategies to manage stress induced by the diagnosis of and living with BMs. While patients more often coped by focusing on positive thinking, maintaining everyday life, trusting the treatment process, and taking one day at a time, family members coped by seeking information, planning for the future, organizing family life, and adapting to a life with uncertainty. These differences persisted over the 4-month interview period, indicating that coping is role-specific: patients’ strategies aimed to manage their illness experience in the present, whereas family members’ strategies focused on anticipating and handling future challenges. Important events, such as new symptoms or medical appointments, reinforced rather than altered these role-specific strategies, emphasizing the distinct ways patients and family members adapt in response to BMs.

Pruyn (1983) reveals that being diagnosed with cancer is a stressor per se. We believe that being additionally diagnosed with BMs further strengthens the negative message of the diagnosis, thereby diminishing patients’ overall sense of well-being. However, people cope better with stressful situations if they have more means by which to manage them. Some means are directed towards removing the cause of stress, while others simply ease the stress. According to Pruyn (1983), to reduce stress, patients usually adopt six common coping strategies: seeking information, seeking support and comfort, attributing causes to events, taking impulsive action, avoiding confrontation, and adopting active coping behaviours. Similar coping strategies were also adopted by the participants in the current study. Patients typically adopted positive thinking and sought support and comfort, while family members primarily sought information as a coping strategy.

Cancer affects not only patients but also their family members; therefore, the family as a unit needs to adjust to a new life situation (Nalbant et al., 2021; Nissen et al., 2016; Woźniak & Iżycki, 2014). The findings reveal that over a 4-month interview period, both patients and their family members faced challenges related to the BMs’ diagnosis, symptom interpretation, complications, and treatment side effects. They had to navigate through various stages of adjustment to the disease. The patients, as well as their family members, felt unprepared, uncertain, and in search of information and support to face these stressors. However, both patients and their family members used different means to reduce stress. Despite the seriousness of the condition, some patients and family members coped with BMs by staying positive, hoping for recovery, and even believing in a cure. Patients tended to place trust in their treatment and healthcare professionals, while family members coped by seeking information and providing support to the patient. For some, planning daily life and gradually adapting to uncertainty helped maintain a sense of normality. These findings indicate that coping strategies ranged from problem-focused to emotion-focused, which we chose to discuss in accordance with Lazarus and Folkman (1984) transactional model of stress and coping.

## Problem-focused coping

A problem-focused coping strategy is adopted when an appraisal can modify or remove the stressful situation (Lazarus & Folkman, 1984). This strategy includes active coping, planning, suppressing competing activities, restraint coping, and seeking instrumental and social support (Carver et al., 1989). In this study, active coping, planning, and seeking instrumental and social support were the most evident problem-focused coping strategies employed by both patients and their family members, although these were applied over time and to varying degrees. The patients often employed avoidance as a coping strategy, consciously diverting attention from their illness to minimize distress. According to Dev et al. (2024), this approach may provide temporary relief but can lead to increased psychological distress over time. By contrast, family members frequently adopted active coping mechanisms, such as seeking information and providing practical support, to manage the uncertainty of the illness trajectory. These proactive strategies are associated with better psychological adjustment and reduced stress (Long et al., 2021).

Active coping, which involves seeking support or making plans, helped participants find meaning through engagement and emotional expression, thereby boosting their self-efficacy and sense of control. Similar findings are also presented in a systematic review by Mardani et al. (2025), exploring qualitative evidence on the coping strategies used by breast cancer survivors to handle the fear of cancer recurrence. The review highlights that actively seeking information and support is a key coping strategy for reducing fear and enhancing understanding of preventive measures.

Changing behaviours, such as asking questions about possible complications, the treatment, its results or side effects, rather than continuing to feel sick and worrying, has a positive impact on patients’ quality of life (Popa-Velea et al., 2017; Velasco et al., 2020). Our study provides evidence that family members were more active than patients and sought information about survival rates and timelines, thus enabling them to perceive their roles from a more meaningful perspective.

Although family members were eager to seek information, similar to the findings of Kårmark et al. (2024), they also wished to maintain normality in their everyday lives. Making plans for the future was a coping strategy that the patients and their family members appraised differently. While some of the patients made plans for one day at a time, family members made long-term life plans. The latter finding mirrors the results of Rachel et al. (2022) study. This coping strategy enabled family members to be proactive, create effective plans, solve problems steadily, and accept the situation to find new solutions.

Another coping strategy was related to the imperative need to seek support and comfort. Both patients and their family members adopted this strategy, primarily by the patients. According to Pruyn (1983), the strategy was an essential means of reducing uncertainties. While both patients and their family members actively sought information, patients primarily sought it for advice and emotional support. In contrast, family members focused on understanding BMs and treatment options to better prepare for the disease’s impact and management. Similarly, Aunan et al. (2021) revealed that prostate cancer survivors need adequate knowledge, both in quantity and quality, to understand what is happening to them and to help them feel in control. Cancer patients are eager to access high-quality, comprehensible, and timely information about their illness, treatments, and symptom management (Evans Webb et al., 2021). Moreover, similar to other studies’ findings (Aunan et al., 2021; Chua et al., 2018; Evans Webb et al., 2021), those of the current study showed that the participants predominantly expressed a need for instrumental support, particularly in the form of information, which highlights their desire to receive the necessary knowledge to cope effectively with the diagnosis, tests and investigations, treatments, and associated side effects. However, in contrast to previous results, our findings reveal that the need to seek information about the diagnosis and treatment was more pronounced among family members and less evident among patients.

Another strategy for active coping, predominantly employed by the patients, was relying on and trusting healthcare professionals, as the patients assessed them to be professional and competent. Similar findings are presented by Gray et al. (2022), who found that participants in their study reported two types of trust: one towards health professionals and another within their social and family networks. The same results were reported in a recently published study by Baum et al. (2025), which explored the meaning of trust along the treatment pathway of women with breast cancer. Trust between healthcare professionals and patients has been shown to improve patients’ quality of life and may enhance clinical outcomes.

## Emotion-focused coping

An emotion-focused coping strategy aims to manage the emotions elicited by a situation rather than amend the situation itself (Lazarus & Folkman, 1984). This strategy encompasses acceptance, self-distraction, positive reframing, self-control, using religion, avoidance, and emotional support (Lazarus & Folkman, 1984). In our study, positive reframing, including positive thinking and maintaining hope, was the most used coping strategy among the patients. The term “positive reframing” means that patients and their family caregivers are optimistic and choose to think positively, which helps them sustain the coping process despite the diagnosis. Similar findings were also revealed in a study by Carrion et al. (2017) exploring the coping strategies used by Latina women with cancer. Optimism is known to have protective effects when dealing with cancer. Early studies involving cancer patients have reported that optimism is associated with better adjustment to cancer, enhanced well-being, and reduced distress while being predictive of treatment challenge acceptance (Carver et al., 1993; Miller et al., 1996).

Hope is a multidimensional and dynamic mental process involving the interaction of motivational energy, strategies, visions, and plans to achieve long-term goals (Nierop van Baalen et al., 2020). Our findings show that, for most participants, especially patients, maintaining hope was based on their belief in treatment, aligning with previous studies that have demonstrated the positive effects of beliefs in curability or hope on the survival of advanced cancer patients (Corn et al., 2022; Yun et al., 2024). However, although patients expressed positivity about the treatment and hoped for a better future, some family members in the current study still expressed a degree of uncertainty about what lies ahead. This sense of insecurity is consistent with findings from an Iranian study that explored fear of cancer recurrence and coping strategies among prostate cancer survivors (Mardani et al., 2023). Fear of cancer recurrence or not surviving is a common concern among both cancer patients and their family members; therefore, interventions addressing this fear in family caregivers could improve the quality of life for both caregivers and cancer survivors (Lamarche et al., 2025).

Avoidance was another emotion-focused coping strategy, particularly used by patients to minimize negative feelings. The patients, in contrast to their family members, chose to distance themselves from reality and avoid negative emotions so that they could carry on with their lives. Despite these challenges, both patients and their family members continuously adapted to a life marked by uncertainty. Coping was not static; it shifted over time, depending on the patient’s condition and the effects of treatment. When patients felt well, family members found it easier to cope with emotions and practical duties. Despite the unpredictability of the illness, both patients and their family members emphasized the importance of adapting to a life with uncertainty. For some, this meant letting go of long-term plans and focusing more on the present. While this shift helped some regain a sense of control, it also brought emotional challenges, such as feeling that life was temporarily “on hold.” Patients found strength in maintaining routines and a sense of agency, while family members coped by reprioritising daily tasks and taking on more practical responsibilities. These adaptive strategies reflect how participants navigated uncertainty through ongoing adjustments in mindset, behaviour, and roles within the family. This finding aligns with Avery et al. (2023), who reported that when participants viewed hardship as uncontrollable, they coped by “letting go”, acknowledging and accepting change to accommodate aspects of the illness seen as unavoidable.

In addition to the coping strategies described by Lazarus and Folkman (1984), an additional emotion-focused coping strategy, self-realization of responsibility towards the family, was identified. For patients with children under the age of 18, providing a sense of normality and continuity for their children became an important coping strategy. Although severe symptoms or treatment side effects stressed them daily, participants recognized their worth and obligations to family members. Consistent with the findings of Nguyen et al. (2023), children, spouses, and the need to maintain family functioning were described as powerful sources of motivation among women with cancer receiving chemotherapy to endure the challenges they faced. This emotion-focused strategy may also be understood in relation to broader concepts such as benefit-finding and post-traumatic growth, where individuals report discovering positive meaning or experiencing personal growth in the midst of adversity. Benefit-finding refers to the identification of positive value amid illness (Cassidy et al., 2014), whereas post-traumatic growth involves more profound shifts in life perspective, relationships, and self-perception (Tedeschi et al., 2018). Previous research has shown that many cancer patients perceive their illness as a catalyst for re-evaluating priorities, strengthening relationships, and deepening their sense of purpose (Almeida et al., 2022; Cafaro et al., 2024; Jansen et al., 2011; Liu et al., 2021). In this context, caring for children and sustaining family life provided patients not only with immediate coping resources but also with opportunities for meaning-making that may foster longer-term resilience.

## Strengths and limitations of the study

One of this study’s strengths is that it provides a comprehensive understanding of coping strategies by including both patients with BMs and their family members, offering unique perspectives from each group. The study highlights the diversity in how individuals manage the uncertainty and emotional burden of BMs, which can be considered a strength. Another strength of the study may be that it examines coping over time (e.g., at diagnosis, two months later, and four months later), allowing for insights into how coping mechanisms evolve throughout the illness trajectory.

A strength of this study is that patients and family members were interviewed separately, which enabled both groups to share their perspectives and coping strategies openly without the influence of each other’s presence. This approach provided rich insights into the distinct coping strategies employed: patients often relied on positive thinking, maintaining everyday life, trusting the treatment process, and focusing on the present, whereas family members tended to cope by seeking information, planning for the future, and organizing family life. However, a limitation of this design is that it did not allow to explore the interaction or alignment of coping strategies within patient—family dyads. Such dynamics may be important, as differences in coping approaches (e.g., patients focusing on the here-and-now while family members plan ahead) could lead to tension or misunderstanding but might also be complementary in managing the challenges of BMs. Furthermore, a couple’s perspectives on the same experiences can provide different insights, potentially leading to alternative findings. Future research could therefore examine coping at the dyadic level to better understand how these strategies intersect, diverge, or mutually support one another in adaptation at different stages of BMs.

Because of the large amount of data generated from the 81 interviews, the interview transcripts were shared among the researchers and analysed separately. This could lead to over- or under-interpretation or even incorrect interpretation of the data. To avoid this, the researchers met several times and discussed the findings until a consensus was reached.

Much of the research literature involving patients with BMs, especially recently published qualitative studies, is almost non-existent. Moreover, longitudinal studies reporting on coping among patients with advanced cancer are scarce (Luu et al., 2024). Therefore, our findings are discussed and supported by those from other studies involving patients with different types of cancers at various stages, rather than patients with BMs, which may limit the transferability of the results to a similar context and patient group. Furthermore, the reliance on some older literature may be regarded as a limitation, reflecting the paucity of recent research specifically addressing patients with BMs that substantiates the present findings, thereby contributing to the qualitative health research literature on this theme.

A potential limitation is that interview transcripts were not returned to participants for verification. However, the follow-up interview design allowed interviewers to revisit earlier responses, enhancing understanding and providing a degree of validation, which may be considered a strength of the study.

Another limitation is that the study’s qualitative nature allows for alternative explanations of the findings; therefore, the findings cannot be considered objective truths. However, the findings suggest subjective descriptions of how participants cope with a BMs diagnosis in their everyday lives, rather than statistically verified knowledge or absolute truths. Interpreting the findings through Lazarus and Folkman (1984) framework may be a limitation, as it could have influenced data interpretation. A different theoretical lens might have led to other insights. Although the study wasn’t initially guided by theory, the framework was applied after data analysis to help explain participants’ coping strategies.

The interviews were moderated by healthcare professionals other than the researchers. This could be a limitation in terms of data analysis, as the researchers could have potentially missed important information provided during interviews that would have strengthened the data analysis. Although initially considered a less-than-ideal means of data collection, some interviews were conducted over the telephone. This may have limited the ability to observe nonverbal cues and build rapport as effectively as in-person interviews do.

Finally, the study participants belonged to the same ethnic group. This could be considered both a limitation and a strength. Participants from the same ethnic group may limit the generalizability of the findings to other populations. However, it offers a more in-depth representation of one setting, which can help researchers compare these findings with those generated in different contexts.

## Clinical implications

Given the distinct coping styles adopted by patients with BMs and their family members, healthcare professionals should design tailored, person- and family-centred interventions that reflect these differences. In practice, this can be operationalized in three ways. First, by incorporating routine assessment of coping strategies and psychological distress, healthcare professionals can identify effective coping mechanisms early and ensure timely referral for further support when needed. Second, tailored psychoeducation and health literacy initiatives, such as structured training programs, can equip both patients and family members with the knowledge and skills to manage daily challenges with greater confidence. These interventions should reflect role-specific needs, for example, supporting patients in maintaining optimism and trust while guiding family members in information seeking, planning, and problem-solving. Third, strengthening healthcare professionals’ communication skills is essential to facilitate clear, compassionate, and family-centred dialogue, helping patients and family members to understand and respect each other’s differing coping approaches. Additionally, expanding awareness of existing support services and developing peer-support groups or community initiatives can provide both emotional encouragement and practical assistance, thereby easing the burden on families. Together, these strategies can help reinforce coping resources and ultimately improve the quality of life and well-being for both patients and their family members.

## Conclusions

This study offers valuable insights into the coping strategies employed by patients with BMs and their family members, as well as the roles, responsibilities, and challenges they face over a 4-month period. Being diagnosed with BMs creates significant stress for both patients and their family members. Our study found that the coping strategies adopted varied, encompassing both problem- and emotion-focused approaches. Revealing the coping strategies of patients with BMs and their family members is vital for the well-being of both groups. Our study revealed that patients and their family members rely on distinct coping strategies: patients often managed their situation by focusing on positive thinking, maintaining everyday routines, trusting the treatment process, and taking one day at a time, while family members coped by seeking information, planning for the future, and organizing family life. These role-specific strategies highlight the different emotional and practical challenges each group faces. By acknowledging and supporting these distinct needs, healthcare systems can foster an environment that strengthens family resilience and enhances the overall quality of care experienced by patients. Comprehensive support initiatives, ranging from clear communication and tailored information to emotional and community-based resources, are essential for alleviating the burden on families and enhancing their coping capacity. Such efforts ultimately benefit both patients and family members, contributing to improved outcomes and a more sustainable approach to cancer care.

## Data Availability

The data supporting the findings of this study are not available in a public repository; however, the data may be made available upon reasonable request and with participants’ consent, upon request from the last author, Tonje Lundeby [tonje.lundeby@medisin.uio.no]. The data are not publicly available due to ethical considerations, including the need for anonymization and the protection of participants’ confidentiality.

## References

[cit0001] Almeida, M., Ramos, C., Maciel, L., Basto-Pereira, M., & Leal, I. (2022). Meaning in life, meaning-making and posttraumatic growth in cancer patients: Systematic review and meta-analysis. *Frontiers in Psychology*, 13, e995981. 10.3389/fpsyg.2022.995981 PMC9784472 36570997

[cit0002] Audulv, Å., Hall, E. O. C., Kneck, Å., Westergren, T., Fegran, L., Pedersen, M. K., Aagaard, H., Dam, K. L., & Ludvigsen, M. S. (2022). Qualitative longitudinal research in health research: A method study. *BMC Medical Research Methodology*, 22(1), 255. 10.1186/s12874-022-01732-4 36182899 PMC9526289

[cit0003] Aunan, S. T., Wallgren, G. C., & Hansen, B. S. (2021). The value of information and support; experiences among patients with prostate cancer. *Journal of Clinical Nursing*, 30(11–12), 1653–20. 10.1111/jocn.15719 33590945

[cit0004] Avery, J., Thomas, R., Howell, D., & Dubouloz Wilner, C.-J. (2023). Empowering cancer survivors in managing their own health: A paradoxical dynamic process of taking and letting go of control. *The Qualitative Health Research*, 33(5), 412–425. 10.1177/10497323231158629 36825869 PMC10126457

[cit0005] Baum, E., Bernhardsgrütter, D., Engst, R., Maurer, C., Ebneter, J., Zenklusen, A., Wartlsteiner, B., Barandun, L., Neher, A., Koller, A., & Kobleder, A. (2025). The meaning of trust along the treatment pathway of women with breast cancer: A mixed-methods study among cancer survivors. *BMC Women’s Health*, 25(1), 25. 10.1186/s12905-024-03540-y 39815217 PMC11737205

[cit0006] Braun, V., & Clarke, V. (2006). Using thematic analysis in psychology. *Qualitative Research in Psychology*, 3(2), 77–101. 10.1191/1478088706qp063oa

[cit0007] Cafaro, V., Rabitti, E., Artioli, G., Costantini, M., De Vincenzo, F., Franzoni, F., Cavuto, S., Bertelli, T., Deledda, G., Piattelli, A., Cardinali, L., De Padova, S., Poli, S., Iuvaro, M. D., Fantoni, G., & DiLeo, S. (2024). Promoting post-traumatic growth in cancer patients: A randomized controlled trial of guided written disclosure. *Frontiers in Psychology*, 15, e1285998. 10.3389/fpsyg.2024.1285998 PMC11008600 38605841

[cit0008] Carrion, I. V., Nedjat-Haiem, F., Macip-Billbe, M., & Black, R. (2017). “I told myself to stay positive” perceptions of coping among latinos with a cancer diagnosis living in the United States. *American Journal of Hospice & Palliative Medicine®*, 34(3), 233–240. 10.1177/1049909115625955 26764346

[cit0009] Carver, C. S., Pozo, C., Harris, S. D., Noriega, V., Scheier, M. F., Robinson, D. S., Ketcham, A. S., Moffat, F. L., Jr., & Clark, K. C. (1993). How coping mediates the effect of optimism on distress: A study of women with early stage breast cancer. *Journal of Personality & Social Psychology*, 65(2), 375–390. 10.1037/0022-3514.65.2.375 8366426

[cit0010] Carver, C. S., Scheier, M. F., & Weintraub, J. K. (1989). Assessing coping strategies: A theoretically based approach. *Journal of Personality & Social Psychology*, 56(2), 267–283. 10.1037/0022-3514.56.2.267 2926629

[cit0011] Cassidy, T., McLaughlin, M., & Giles, M. (2014). Benefit finding in response to general life stress: Measurement and correlates. *Health Psychology and Behavioral Medicine*, 2(1), 268–282. 10.1080/21642850.2014.889570 25750781 PMC4346032

[cit0012] Chua, G. P., Tan, H. K., & Gandhi, M. (2018). What information do cancer patients want and how well are their needs being met? *Ecancermedicalscience*, 12, 873. 10.3332/ecancer.2018.873 30483353 PMC6214674

[cit0013] Corn, B. W., Feldman, D. B., Hull, J. G., O’Rourke, M. A., & Bakitas, M. A. (2022). Dispositional hope as a potential outcome parameter among patients with advanced malignancy: An analysis of the ENABLE database. *Cancer*, 128(2), 401–409. 10.1002/cncr.33907 34613617 PMC10008020

[cit0014] Dev, R., Agosta, M., Fellman, B., Reddy, A., Baldwin, S., Arthur, J., Haider, A., Carmack, C., Hui, D., & Bruera, E. (2024). Coping strategies and associated symptom burden among patients with advanced cancer. *The Oncologist*, 29(2), 166–175. 10.1093/oncolo/oyad253 37669020 PMC10836315

[cit0015] Dhanabhakyam, M., & Sarath, M. (2023). Psychological wellbeing: A systematic literature review. *International Journal of Advanced Research in Science, Communication and Technology*, 3(1), 603–607. 10.48175/IJARSCT-8345

[cit0016] Evans Webb, M., Murray, E., Younger, Z. W., Goodfellow, H., & Ross, J. (2021). The supportive care needs of cancer patients: A systematic review. *Journal of Cancer Education*, 36(5), 899–908. 10.1007/s13187-020-01941-9 33492650 PMC8523012

[cit0017] Fares, J., Petrosyan, E., Dmello, C., Lukas, R. V., Stupp, R., & Lesniak, M. S. (2025). Rethinking metastatic brain cancer as a CNS disease. *The Lancet Oncology*, 26(2), e111–e121. 10.1016/S1470-2045(24)00430-3 39914421

[cit0018] Folkman, S., & Moskowitz, J. T. (2004). Coping: Pitfalls and promise. *The Annual Review of Psychology*, 55(1), 745–774. 10.1146/annurev.psych.55.090902.141456 14744233

[cit0019] Gray, T. F., Allgood, S. J., Nolan, M. T., Gallo, J. J., Han, H.-R., Clayman, M. L., Budhathoki, C., Lansey, D. G., & Wenzel, J. (2022). “It all depends”: Patient and decision partner experiences in cancer clinical trial decision-making. *The Qualitative Health Research*, 32(6), 887–901. 10.1177/10497323221083355 35343318

[cit0020] Greer, J. A., Moy, B., El-Jawahri, A., Jackson, V. A., Kamdar, M., Jacobsen, J., Lindvall, C., Shin, J. A., Rinaldi, S., Carlson, H. A., Sousa, A., Gallagher, E. R., Li, Z., Moran, S., Ruddy, M., Anand, M. V., Carp, J. E., & Temel, J. S. (2022). Randomized trial of a palliative care intervention to improve end-of-life care discussions in patients with metastatic breast cancer. *Journal of the National Comprehensive Cancer Network*, 20(2), 136–143. 10.6004/jnccn.2021.7040 35130492 PMC8830600

[cit0021] Harel, K., Czamanski-Cohen, J., Cohen, M., Caspi, O., & Weihs, K. L. (2025). Coping, emotional processing, and cancer-related symptoms in breast cancer survivors: Cross-sectional secondary analysis of the REPAT study. *Psycho-Oncology*, 34(2), e70094. 10.1002/pon.70094 39891317 PMC11785827

[cit0022] Harrison, R. A., Tang, M., Shih, K. K., Khan, M., Pham, L., De Moraes, A. R., O’Brien, B. J., Bassett, R., & Bruera, E. (2024). Characterization of patients with brain metastases referred to palliative care. *BMC Palliative Care*, 23(1), 13. 10.1186/s12904-023-01320-3 38212765 PMC10782691

[cit0023] Hernández, R., Calderon, C., Carmona-Bayonas, A., Rodríguez Capote, A., Jara, C., Padilla Álvarez, A., Gómez-Camacho, M. L. N., Beato, C., Castelo, B., Majem, M., Muñoz, M. D. M., Ivars, A., Mangas-Izquierdo, M., Rogado-Revuelta, J., & Jimenez-Fonseca, P. (2019). Differences in coping strategies among young adults and the elderly with cancer. *Psychogeriatrics*, 19(5), 426–434. 10.1111/psyg.12420 30723983

[cit0024] Ismael, N., Jaber, A., Malkawi, S., Al Awady, S., & Ismael, T. (2024). Exploring coping strategies among caregivers of children who have survived paediatric cancer in Jordan. *BMJ Paediatrics Open*, 8(1), e002453. 10.1136/bmjpo-2023-002453 38604770 PMC11015291

[cit0025] Jansen, L., Hoffmeister, M., Chang-Claude, J., Brenner, H., & Arndt, V. (2011). Benefit finding and post-traumatic growth in long-term colorectal cancer survivors: Prevalence, determinants, and associations with quality of life. *British Journal of Cancer*, 105(8), 1158–1165. 10.1038/bjc.2011.335 21878935 PMC3208486

[cit0026] Kaiser, U., Vehling-Kaiser, U., Kück, F., Mechie, N.-C., Hoffmann, A., & Kaiser, F. (2020). Use of symptom-focused oncological cancer therapies in hospices: A retrospective analysis. *BMC Palliative Care*, 19(1), 140. 10.1186/s12904-020-00648-4 32919468 PMC7488695

[cit0027] Kang, Y., & Son, H. (2019). Age differences in the coping strategies of patients with colorectal cancer. *Cancer Nursing*, 42(4), 286–294. 10.1097/ncc.0000000000000604 29757771

[cit0028] Kårmark, S., Malmström, M., & Kristensson, J. (2024). Together but still alone - a qualitative study exploring how family members of persons with incurable oesophageal or gastric cancer manage everyday life. *BMC Palliative Care*, 23(1), 249. 10.1186/s12904-024-01576-3 39462393 PMC11515144

[cit0029] Kelly, M. M., Martin-Peters, T., & Farber, J. S. (2024). Secondary data analysis: Using existing data to answer new questions. *Journal of Pediatric Health Care*, 38(4), 615–618. 10.1016/j.pedhc.2024.03.005 38556962

[cit0030] Knott, E., Rao, A. H., Summers, K., & Teeger, C. (2022). Interviews in the social sciences. *Nature Reviews Methods Primers*, 2(1), 73. 10.1038/s43586-022-00150-6

[cit0031] Koekkoek, J. A. F., van der Meer, P. B., Pace, A., Hertler, C., Harrison, R., Leeper, H. E., Forst, D. A., Jalali, R., Oliver, K., Philip, J., Taphoorn, M. J. B., Dirven, L., & Walbert, T. (2023). Palliative care and end-of-life care in adults with malignant brain tumors. *Neuro-Oncology*, 25(3), 447–456. 10.1093/neuonc/noac216 36271873 PMC10013651

[cit0032] Lamarche, J., Nissim, R., Avery, J., Wong, J., Maheu, C., Lambert, S. D., Laizner, A. M., Jones, J., Esplen, M. J., & Lebel, S. (2025). It is time to address fear of cancer recurrence in family caregivers: Feasibility and acceptability of a randomized pilot study of the family caregiver version of the fear of recurrence therapy (FC-FORT). *Psycho-Oncology*, 34(2), e70084. 10.1002/pon.70084 39887474 PMC11779570

[cit0033] Lambert, S., Moodie, E. E. M., McCusker, J., Lokhorst, M., Harris, C., Langmuir, T., Belzile, E., Laizner, A. M., Brahim, L. O., Wasserman, S., Chehayeb, S., Vickers, M., Duncan, L., Esplen, M. J., Maheu, C., Howell, D., & de Raad, M. (2025). Translating evidence-based self-management interventions using a stepped-care approach for patients with cancer and their caregivers: A pilot sequential multiple assignment randomized trial design. *Psycho-Oncology*, 34(1), e70043. 10.1002/pon.70043 39763142 PMC11704335

[cit0034] Lazarus, R. S., & Folkman, S. (1984). *Stress, appraisal, and coping*. Springer.

[cit0035] Lincoln, Y. S., & Guba, E. G. (1985). *Naturalistic inquiry*. Sage.

[cit0036] Liu, Z., Thong, M. S. Y., Doege, D., Koch-Gallenkamp, L., Bertram, H., Eberle, A., Holleczek, B., Waldmann, A., Zeissig, S. R., Pritzkuleit, R., Brenner, H., & Arndt, V. (2021). Prevalence of benefit finding and posttraumatic growth in long-term cancer survivors: Results from a multi-regional population-based survey in Germany. *British Journal of Cancer*, 125(6), 877–883. 10.1038/s41416-021-01473-z 34215852 PMC8437934

[cit0037] Long, N. X., Ngoc, N. B., Phung, T. T., Linh, D. T. D., Anh, T. N., Hung, N. V., Thang, N. T., Lan, N. T. M., Trang, V. T., Thuong, N. H., Van Hieu, N., & Van Minh, H. (2021). Coping strategies and social support among caregivers of patients with cancer: A cross-sectional study in Vietnam. *AIMS Public Health*, 8(1), 1–14. 10.3934/publichealth.2021001 33575403 PMC7870390

[cit0038] Luu, K. L., Mager, P., Nieboer, D., Witkamp, F. E., Jabbarian, L. J., Payne, S., Groenvold, M., Pollock, K., Miccinesi, G., Deliens, L., van Delden, J. J. M., van der Heide, A., Korfage, I. J., & Rietjens, J. A. C. (2024). Coping strategies of patients with advanced lung or colorectal cancer over time: Insights from the international ACTION study. *Psycho-Oncology*, 33(10), e9315. 10.1002/pon.9315 39363364

[cit0039] Ma, H., Yang, Y., Li, Y., Cariola, L., & Gillanders, D. (2025). Effectiveness of psychological interventions in improving relationship functioning among couples coping with prostate cancer: A systematic review and meta-analysis. *Psycho-Oncology*, 34(1), e70080. 10.1002/pon.70080 39804293 PMC11728261

[cit0040] Maqbool, T., Agarwal, A., Sium, A., Trang, A., Chung, C., & Papadakos, J. (2017). Informational and supportive care needs of brain metastases patients and caregivers: A systematic review. *Journal of Cancer Education*, 32(4), 914–923. 10.1007/s13187-016-1030-5 27041700

[cit0041] Mardani, A., Farahani, M. A., Khachian, A., Maleki, M., & Vaismoradi, M. (2024). Qualitative exploration of sexual dysfunction and associated coping strategies among Iranian prostate cancer survivors. *Supportive Care in Cancer*, 32(6), 360. 10.1007/s00520-024-08548-6 38753060

[cit0042] Mardani, A., Farahani, M. A., Khachian, A., & Vaismoradi, M. (2023). Fear of cancer recurrence and coping strategies among prostate cancer survivors: A qualitative study. *Current Oncology*, 30(7), 6720–6733. 10.3390/curroncol30070493 37504353 PMC10378434

[cit0043] Mardani, A., Maleki, M., Hanifi, N., Turunen, H., & Vaismoradi, M. (2025). Coping strategies for fear of cancer recurrence among breast cancer survivors: A systematic review and thematic synthesis of qualitative studies. *Supportive Care in Cancer*, 33(6), 459. 10.1007/s00520-025-09503-9 40341433

[cit0044] Miller, D. L., Manne, S. L., Taylor, K., Keates, J., & Dougherty, J. (1996). Psychological distress and well-being in advanced cancer: The effects of optimism and coping. *Journal of Clinical Psychology in Medical Settings*, 3(2), 115–130. 10.1007/bf01996132 24226639

[cit0045] Mitchell, D. K., Kwon, H. J., Kubica, P. A., Huff, W. X., O’Regan, R., & Dey, M. (2022). Brain metastases: An update on the multi-disciplinary approach of clinical management. *Neurochirurgie*, 68(1), 69–85. 10.1016/j.neuchi.2021.04.001 33864773 PMC8514593

[cit0046] Morse, J. M. (2015). Critical analysis of strategies for determining rigor in qualitative inquiry. *The Qualitative Health Research*, 25(9), 1212–1222. 10.1177/1049732315588501 26184336

[cit0047] Nalbant, B., Karger, A., & Zimmermann, T. (2021). Cancer and relationship dissolution: Perspective of partners of cancer patients. *Frontiers in Psychology*, 12, 624902. 10.3389/fpsyg.2021.624902 34093310 PMC8177048

[cit0048] Nguyen, K. T., Vu, N. T. H., Tran, M. T. T., & Chan, C. W. H. (2023). A qualitative study on stress, coping strategies and feasibility of music intervention among women with cancer receiving chemotherapy during COVID-19 pandemic in Vietnam. *Scientific Reports*, 13(1), 542. 10.1038/s41598-023-27654-9 36631561 PMC9832410

[cit0049] Nierop van Baalen, C., Grypdonck, M., van Hecke, A., & Verhaeghe, S. (2020). Associated factors of hope in cancer patients during treatment: A systematic literature review. *Journal of Advanced Nursing*, 76(7), 1520–1537. 10.1111/jan.14344 32133663

[cit0050] Nissen, K. G., Trevino, K., Lange, T., & Prigerson, H. G. (2016). Family relationships and psychosocial dysfunction among family caregivers of patients with advanced cancer. *Journal of Pain and Symptom Management*, 52(6), 841–849. 10.1016/j.jpainsymman.2016.07.006 27521285 PMC5497710

[cit0051] Oppegaard, K. R., Dunn, L. B., Kober, K. M., Mackin, L., Hammer, M. J., Conley, Y. P., Levine, J., & Miaskowski, C. (2020). Gender differences in the use of engagement and disengagement coping strategies in patients with cancer receiving chemotherapy. *Oncology Nursing Forum*, 47(5), 586–594. 10.1188/20.Onf.586-594 32830804 PMC10788967

[cit0052] Pinquart, M., & Duberstein, P. R. (2010). Depression and cancer mortality: A meta-analysis. *Psychological Medicine*, 40(11), 1797–1810. 10.1017/S0033291709992285 20085667 PMC2935927

[cit0053] Popa-Velea, O., Diaconescu, L., Jidveian Popescu, M., & Truţescu, C. (2017). Resilience and active coping style: Effects on the self-reported quality of life in cancer patients. *International Journal of Psychiatry in Medicine*, 52(2), 124–136. 10.1177/0091217417720895 28792288

[cit0054] Pruyn, J. F. A. (1983). Coping with stress in cancer patients. *Patient Education & Counseling*, 5(2), 57–62. 10.1016/0738-3991(83)90002-2 10264444

[cit0055] Rachel, K., Rukundo, G., Mutto, M., Orem, J., Niyonzima, N., & Kizito, S. (2022). Coping and caregiving-satisfaction among caregivers of patients with cancer at the Uganda Cancer Institute and Mbarara Regional Referral Hospital in Uganda. *Open Journal of Medical Psychology*, 11, 170–181. 10.4236/ojmp.2022.113013

[cit0056] Saunders, B., Sim, J., Kingstone, T., Baker, S., Waterfield, J., Bartlam, B., Burroughs, H., & Jinks, C. (2018). Saturation in qualitative research: Exploring its conceptualization and operationalization. *Quality and Quantity*, 52(4), 1893–1907. 10.1007/s11135-017-0574-8 29937585 PMC5993836

[cit0057] Tedeschi, R. G., Shakespeare-Finch, J., & Taku, K. (2018). *Posttraumatic growth: Theory, research, and applications*. Routledge.

[cit0058] Triebel, K. L., Gerstenecker, A., Meneses, K., Fiveash, J. B., Meyers, C. A., Cutter, G., Marson, D. C., Martin, R. C., Eakin, A., Watts, O., & Nabors, L. B. (2015). Capacity of patients with brain metastases to make treatment decisions. *Psycho-Oncology*, 24(11), 1448–1455. 10.1002/pon.3753 25613039 PMC4512930

[cit0059] Unsar, S., Erol, O., & Ozdemir, O. (2021). Caregiving burden, depression, and anxiety in family caregivers of patients with cancer. *European Journal of Oncology Nursing*, 50, 101882. 10.1016/j.ejon.2020.101882 33421929

[cit0060] Velasco, L., Gutiérrez Hermoso, L., Alcocer Castillejos, N., Quiroz Friedman, P., Peñacoba, C., Catalá, P., & Sánchez-Román, S. (2020). Association between quality of life and positive coping strategies in breast cancer patients. *Women & Health*, 60(9), 1063–1069. 10.1080/03630242.2020.1802398 32752953

[cit0061] Wei, C. W., Liang, S. Y., Chin, C. H., Lin, H. C., & Rosenberg, J. (2023). Change trajectory of symptom distress, coping strategies, and spiritual wellbeing in colorectal cancer patients undergoing chemotherapy. *Healthcare (Basel)*, 11(6), e857. 10.3390/healthcare11060857 PMC10047921 36981514

[cit0062] World Medical Association. (2025). World Medical Association declaration of Helsinki: Ethical principles for medical research involving human participants. *JAMA*, 333(1), 71–74. 10.1001/jama.2024.21972 39425955

[cit0063] Woźniak, K., & Iżycki, D. (2014). Cancer: A family at risk. *Przegląd Menopauzalny* [Menopause Review], 4(4), 253–261. 10.5114/pm.2014.45002 PMC4520372 26327863

[cit0064] Yi, X., & Kase, H. (2023). Coping strategies among middle-aged and older cancer survivors in Japan: A qualitative study. *International Social Work*, 66(3), 904–918. 10.1177/00208728211068933

[cit0065] Yildirim, D., Çiriş Yildiz, C., Ozdemir, F. A., Harman Özdoğan, M., & Can, G. (2024). Effects of a mindfulness-based stress reduction program on stress, depression, and psychological well-being in patients with cancer: A single-blinded randomized controlled trial. *Cancer Nursing*, 47(2), E84–E92. 10.1097/NCC.0000000000001173 36480346

[cit0066] Yip, C., Valilis, E., Prichett, L., Burdalski, C., & Feliciano, J. (2021). EPR21-040. Prescribing patterns at the end of life in lung cancer. *Journal of the National Comprehensive Cancer Network*, 19(3.5), EPR21–040–EPR021–040. 10.6004/jnccn.2020.7790

[cit0067] Yun, J.-Y., Jung, J. Y., Keam, B., Lee, N.-R., Kang, J. H., Kim, Y. J., Shim, H.-J., Jung, K. H., Koh, S.-J., Ryu, H., Yoo, S. H., Kang, E., & Yun, Y. H. (2024). Depression, performance status, and discontinued treatment mediate an association of curability belief with prognosis in advanced cancer patients. *Scientific Reports*, 14(1), 29098. 10.1038/s41598-024-80687-6 39582048 PMC11586441

